# Constitutive Stringent Response Restores Viability of *Bacillus subtilis* Lacking Structural Maintenance of Chromosome Protein

**DOI:** 10.1371/journal.pone.0142308

**Published:** 2015-11-05

**Authors:** Camille Benoist, Cyprien Guérin, Philippe Noirot, Etienne Dervyn

**Affiliations:** 1 INRA, UMR1319 Micalis, 78350, Jouy-en-Josas, France; 2 AgroParisTech, UMR Micalis 1319, 78350, Jouy-en-Josas, France; 3 Mathématiques et Informatique Appliquées du Génome à l’Environnement, UR1404, INRA, Domaine de Vilvert, 78350, Jouy-en-Josas, France; University of Groningen, Groningen Institute for Biomolecular Sciences and Biotechnology, NETHERLANDS

## Abstract

*Bacillus subtilis* mutants lacking the SMC-ScpAB complex are severely impaired for chromosome condensation and partitioning, DNA repair, and cells are not viable under standard laboratory conditions. We isolated suppressor mutations that restored the capacity of a *smc* deletion mutant (*Δsmc*) to grow under standard conditions. These suppressor mutations reduced chromosome segregation defects and abrogated hypersensitivity to gyrase inhibitors of *Δsmc*. Three suppressor mutations were mapped in genes involved in tRNA aminoacylation and maturation pathways. A transcriptomic survey of isolated suppressor mutations pointed to a potential link between suppression of *Δsmc* and induction of the stringent response. This link was confirmed by (p)ppGpp quantification which indicated a constitutive induction of the stringent response in multiple suppressor strains. Furthermore, sublethal concentrations of arginine hydroxamate (RHX), a potent inducer of stringent response, restored growth of *Δsmc* under non permissive conditions. We showed that production of (p)ppGpp alone was sufficient to suppress the thermosensitivity exhibited by the *Δsmc* mutant. Our findings shed new light on the coordination between chromosome dynamics mediated by SMC-ScpAB and other cellular processes during rapid bacterial growth.

## Introduction

Efficient chromosome organization and segregation, as well as maintenance of genome integrity, are essential for accurate transmission of hereditary genetic information. Proteins from the SMC family are key players in chromosome dynamics that involve chromosome condensation and segregation, cohesion of sister chromatids and DNA repair [1, 2]. Genes encoding SMC proteins have been found in every sequenced eukaryote to date and in most prokaryote genomes [3, 4]. SMC proteins share a common architecture with a globular domain carrying an ABC-type ATPase activity and a “hinge” domain separated by a long antiparallel coiled-coil region. SMC dimers form via interactions between two hinge domains, and higher level intermolecular interactions can take place via the globular domains in an ATP dependant manner. SMC dimers also interact with non-SMC proteins such as the kleisin-like proteins [4]. The inactivation of non-SMC proteins have a “SMC-like” phenotype demonstrating they are key factors for the activity of the SMC complexes.

Whereas eukaryotic genomes encode several functionally specialized SMC complexes [4], a single SMC complex is found in most bacteria. To date, three different types of SMC complexes have been identified in bacteria: (i) SMC-ScpAB [5, 6] broadly represented in eubacteria and archaea, (ii) MukBEF found mainly in enterobacteria [7], and (iii) MksBEF [8] recently described in *Pseudomonas aeruginosa*.

Intriguingly, while the SMC complex is dispensable for cell viability in some bacteria such as *Deinococcus radiodurans* and *Staphylococcus aureus* [9, 10], the growth of *Escherichia coli* MukBEF- and *Bacillus subtilis* SMC-ScpAB-depleted mutants is restricted to conditions allowing slow growth (*e*.*g*. minimal mineral medium or temperatures below 25°C) [11–13]. Even under these permissive conditions, the *smc*-null mutants display aberrant phenotypes including nucleoid decondensation, segregation defects (anucleated cells, “guillotine” phenotypes where a segment of chromosome remains trapped in the division septum), hypersensitivity to gyrase inhibitors [14, 15] and to DNA damaging agents [16] and origin segregation defects [17].

In *E*. *coli* different mutations in *topA*, encoding DNA topoisomerase I, suppress the global deficiencies in a *mukB*-null strain [18], implying a major role of MukBEF in the maintenance of DNA supercoiling and chromosome condensation; it is considered as a chromosomal organizer [19]. In *B*. *subtilis*, decreasing the expression of DNA topoisomerase I or increasing expression of DNA topoisomerase IV, encoded by *parCD*, substantially restored chromosome partitioning and condensation in a *smc*-null strain under permissive conditions but failed to restore viability under non permissive conditions [15, 20]. Thus altered levels of topoisomerases partially correcting defects in chromosome supercoiling and condensation appear insufficient to restore viability of the *smc*-null mutant under non permissive conditions, suggesting that *B*. *subtilis* SMC-ScpAB complex may have additional yet unknown functions.

Supporting this idea, SMC complexes are found preferentially located in the vicinity of the replication origin (*ori*) of the chromosome, suggesting that they may be involved in polar condensation of DNA from the *ori* region, [21] and directly promoting efficient segregation of the origin [22]. In *B*. *subtilis* and *Streptococcus pneumonia*, the localization of SMC complexes is dependent on the partitioning ParB protein (Spo0J in *B*. *subtilis*) and its binding to the *parS* sites located near the replication origin [23–26]. However in absence of Spo0J or *parS* sites, only moderate segregation defects and no sensitivity to temperature above 25°C are observed, supporting the notion that SMC-ScpAB functions are not restricted to these so-called “condensation centers” [24, 27]. Notably, SMC appears to be particularly enriched at highly transcribed regions of the *B*. *subtilis* chromosome [25], but the physiological role of SMC complexes at these sites remains unknown.

Considering the highly pleiotropic phenotypes exhibited by *B*. *subtilis* in absence of SMC, we investigated whether cell death under non permissive conditions is due to a synergistic effect of interdependent problems (*e*.*g*. condensation, segregation, DNA repair) or to another critical process that remains to be identified. To this end, we characterized suppressor mutations that restore viability of a *Δsmc* mutant under non permissive conditions. We discovered that the constitutive induction of the stringent response either by mutations or by amino acid analogs fully restored cell viability and considerably reduced defects in chromosome segregation and condensation as already observed [17]. In addition, we showed that the expression of an *E*. *coli* RelA protein constitutively producing (p)ppGpp restored full cell viability, resistance to gyrase inhibitors, and partially reduced the proportion of anucleate cells.

Considering that i) the stringent response represses rRNA synthesis [28] and slows down DNA replication forks [29] ii) the identification of the suppressors mutations and iii) the localization of the SMC complex in highly transcribed regions (including tRNA operon), we discuss the possibility that the SMC-ScpAB complex could be required to efficiently replicate, segregate and maintain the integrity of these specific rRNA loci close to the origin. We propose that SMC-ScpAB acts to coordinate transcription and replication/segregation, ensuring the efficient organization of newly replicated DNA.

## Results

### Isolation of suppressors restoring the viability of the *Δsmc* mutant under non permissive conditions

The *Δsmc* mutant grows in minimal medium at least up to 37°C and in rich medium up to 25°C but does not grow on rich medium at 37°C. To isolate spontaneous suppressor mutations restoring growth of the Δ*smc* mutant, 10 independent cultures were grown in minimal medium at 30°C, numerated on this medium, and plated on the non-permissive LB medium at 37°C. In this treatment, the cells are subjected to two simultaneous shifts in temperature and medium richness. The proportion of cells that survived this treatment was ~10^−4^, a value unexpectedly high relative to the proportion of mutants in a similar assay with another essential gene (~10^−8^) [30]. To rule out that *Δsmc* could display a hyper-mutator phenotype, we compared the frequency of spontaneous mutations occurring in the rifampicin binding site of the RNA polymerase β subunit [31] and conferring resistance to rifampicin (Rif^R^). Similar frequencies of Rif^R^ cells were detected in the wild type and isogenic *Δsmc* strains (respectively 1.0 10^−8^ +/- 0.1 10^−8^ and 0.8 10^−8^ +/- 0.4 10^−8^), indicating that the loss of SMC complex does not cause any hyper-mutator phenotype. This finding suggests that the elevated frequency of *Δsmc* suppressors is likely due to the presence of multiple genes that can confer the suppressor phenotype.

For each of the 10 independent cultures, two suppressor colonies were isolated on rich medium at 37°C for further analysis. We compared the phenotypes of the 20 suppressor strains with wild type and *Δsmc* strains for the following criteria (i) sensitivity to gyrase inhibitors (coumermycin A1 and nalidixic acid, targeting the gyrase subunits GyrB and GyrA respectively), (ii) ability to grow at 51°C, and (iii) size of colonies formed on rich medium. These criteria allowed us to establish seven phenotypic classes of mutants (Table 1). In each of the tested conditions, the phenotypes exhibited by suppressor strains ranged between extremes represented by the wild type and *Δsmc* mutant phenotypes, with the exception of sensitivity to nalidixic acid, where each selected suppressor was surprisingly more resistant than *Δsmc* and wild type. The presence of suppressor mutations in the genes encoding DNA gyrase, DNA topisomerases I, III, and IV was tested by measuring the genetic linkage between suppressors and topoisomerase genes or by direct sequencing of promoter and coding regions for the *gyrAB* genes. None of the suppressor strains carried mutations within or genetically linked to DNA topoisomerase genes, leaving the increased resistance to nalidixic acid unexplained.

**Table 1:** Classification of *Δsmc* suppressor mutants.

Classes	Strain	Colony size	LB 51°C	MC40 ng/ml	Coum0.5 μg/ml	Nal. acid1 μg/ml
I	s34	M	**0.3**	**0.002**	**0.5**	**0.5**
II	s35, s36, s37	L	**0.5**	**0.003**	**0.0009**	**0.6**
III	s38, s40, s41	S	**0.0001**	**0.008**	**0.6**	**0.8**
IV	s44, s46, s47, s48, s52, s53, s54	S	**0.4**	**0.008**	**0.5**	**0.09**
V	s43, s49	S	**0.009**	**0.006**	**0.05**	**0.09**
VI	s42, s55	S	**0.5**	**0.0009**	**0.5**	**0.9**
VII	s33, s39	L	**0.0001**	**0.001**	**0.5**	**0.8**
	***Δ smc***	S	**0.00001**	**0.0004**	**0.0008**	**0.002**
	**168**	L	**1**	**0.09**	**1**	**0.004**

The twenty suppressors were grouped in seven different classes based on phenotypic analyses. Colony size was estimated after 72h at 23°C on rich medium: Colony size similar to wild type is large (L), similar to Δ*smc* is small (S), and intermediate is medium (M). Plating efficiencies were determined from cells in exponential growth phase. Survival assays on mitomycin C (MC), coumermycin A1 (Coum), and nalidixic acid (Nal. Acid) were performed on rich medium at 23°C with the indicated concentrations. The ratio between the numbers of colonies obtained in each condition relative to the number of viable cells on rich medium at 23°C was calculated. The value in the table corresponds to the means of the ratio for at least two independent experiments per strains. Five suppressors formed colonies almost as large as wild type colonies. Three of these (class II: s35, s36, s37) were able to grow at 51°C but still demonstrated a high sensitivity to coumermycin A1, unlike the remaining two (class VII: s33, s39). Among suppressors forming small colonies, five were unable to grow at 51°C on rich medium and were separated into two classes to their resistance (class III: s38, s40, s41) or sensitivity (class V: s43, s49) to gyrase inhibitors. The final suppressors (nine of the original twenty) grew at 51°C, formed small colonies, were resistant to coumermycin A1 and showed either an intermediate sensitivity (class IV: s44, s46, s47, s48, s52, s53, s54) or high resistance (class VI: s42, s55) to nalidixic acid. Suppressor s34 showed nearly the same phenotype as the suppressors belonging to the class VI, although it appeared earlier and formed larger colonies on plates, earning its separation from the other suppressors in the class I.

### Three suppressor mutations affect tRNA modification and aminoacylation pathways

The genome of one representative strain from each class of suppressors (i.e. s34, s35, s38, s46, s43, s42, s33) was sequenced by high-throughput sequencing (SOLiD). The short reads (35 bases) were projected onto the reference genome to identify the potential suppressor mutations (see Materials and Methods). This analysis did not detect single bases deletions/insertions and did not cover repeated genes (*e*.*g*. rDNA and tDNA), possibly explaining the two strains without any detected mutation (S1 Table). Two strains, s33 and s35, contained multiple mutations and were not further characterized. Three strains carried a single mutation each in a different gene: s38, substitution in AspS (V_432_G); s42, substitution in YwlC (P_64_L); and s34, a deletion causing the removal of 32 amino acids (Δ232–264) in YlbM. These two last mutations were backcrossed in the wild type 168 background and then combined with *Δsmc*, as described in Materials and Methods. Each reconstructed double mutant strain exhibited phenotypes indistinguishable from the original suppressor strain (Table 2), indicating that the identified mutations were causing the suppression. Furthermore, we deleted entirely the *ywlC* and *ylbM* genes, which are dispensable, unlike *aspS*, and combined these deletions with *Δsmc*. We found that both *ΔywlC* and *ΔylbM* restored the viability of the *Δsmc* mutant under non permissive conditions, suggesting that the spontaneous suppressor mutations entail a loss of YlbM or YwlC functions.

**Table 2:** Percentage of anucleate cells and growth rate of suppressors cultivated in slow or fast growth condition.

Strain	% of anucleated cells (number of cells)		Growth rate in minutes	
	MM 30°C	LB 37°C	23°C	37°C
Wild type	<0.3 (345)	<0.4 (263)	84	21
Δ*smc*	8.7 (448)	NA	241	NA
Δ*smc ylbM*(Δ232–264) (s34)	4.1 (417)	13.8 (420)	203	58
Δ*smc aspS*(V_432_>G) (s38)	4.7 (498)	6.7 (520)	141	70
Δ*smc ywlC*(P_64_>L) (s42)	3.7 (267)	6.5 (352)	246	71
*ylbM*(Δ232–264) (s34)	<0.5 (200)	<0.5 (200)	NA	36
*ywlC*(P_64_>L) (s42)	<0.5 (200)	<0.5 (200)	NA	66

Cells were grown in LB at 37°C (non permissive) and in MM at 30°C (permissive) to exponential growth phase (OD_600nm_ between 0.3 and 0.6). The percentage of anucleate cells was measured after staining of DNA with DAPI and membranes with FM4-64FX and visualization of cells by microscopy. Number of examined cells is shown in parentheses. Generation time during exponential growth phase of the different strains has been estimated from monitoring optical density (OD_600nm_) of cultures in rich medium at 23°C and 37°C. To determine ratio of colonies formed per OD_600nm_ unit, cells were grown in rich medium at 23°C or 37°C up to exponential growth phase (OD_600nm_ between 0.3 and 0.6) and then spread on LB plate and incubated 3 to 4 days at 23°C. NA: not applicable. The average from a minimum of two independent experiments is shown.

Remarkably, the three proteins mutated in the suppressor strains can be functionally associated with tRNA modification and aminoacylation. YwlC (also named TscA) belongs to the universal protein family YrdC/Sua5, which is required for threonyl-carbamoyl-adenosine (t(6)A) modification of some tRNAs at position 37 [32, 33]. AspS is an aminoacyl-tRNA synthetase that charges the tRNA^Asp^ with aspartic acid [34]. YlbM is a predicted HIGH motif-containing aminoacyl-tRNA-synthetase related to nucleotidyl transferases [35], and the neighborhood of *ylbM* and *rpmF* (encoding the 50S ribosomal protein L32) is highly conserved throughout Firmicutes genomes, suggesting an evolutionary conserved association with translation. To obtain further information concerning its function, an YlbM-SPA tag fusion protein was constructed [36]. This fusion is fully functional as the phenotypes of *Δsmc ylbM-SPA* and the *Δsmc* strains are the same and are clearly different from those of the *Δsmc ΔylbM* strain (see Materials and Methods). *The* SPA-tagged YlbM was purified by a tandem affinity procedure allowing to co-purify specific proteins partners [37]. A total of 19 potential proteins partners were identified (S2 Table), including 6 tRNA-synthetases. Although the nature and function of these potential interactions remain unknown, this finding further supports a potential role of YlbM in the charged tRNA biosynthesis.

**Fig 1:**
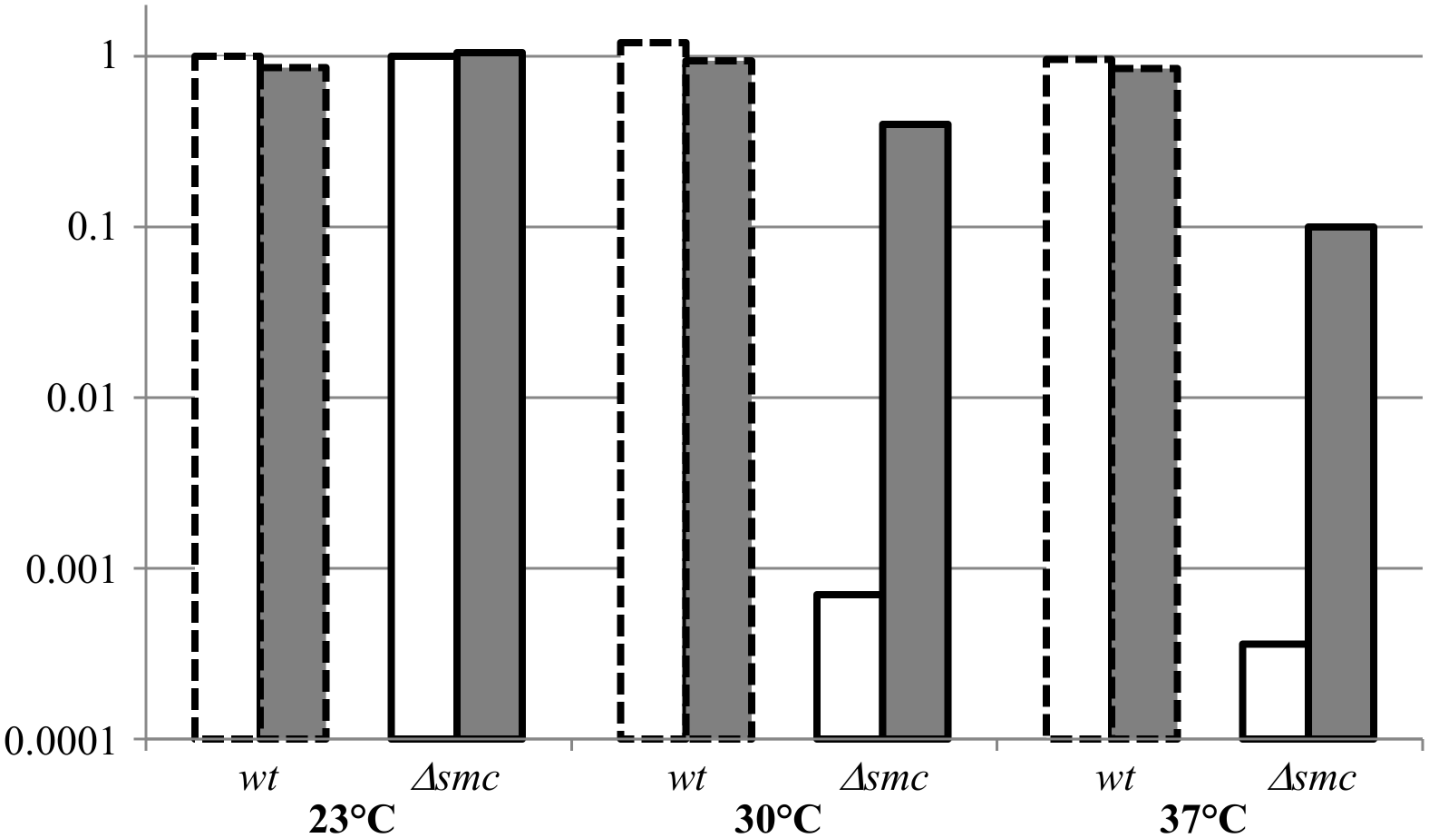
Plate efficiency with or without mupirocin at different temperatures. Wild type and Δ*smc* cells were grown in permissive condition (LB at 23°C), diluted and spread on LB plates in the presence and absence of mupirocin 20ng/ml and incubated at permissive (23°C) and non permissive (30°C, 37°C) temperature. We calculated the ratio of colony forming units in the test condition versus the number of colony forming units in permissive condition (LB at 23°C without mupirocin). The figure show the data for the wild type strain (dotted line)and the Δ*smc* strain (full line) without (white) or with mupirocin (grey) at different temperatures.

All together the data indicate a link between suppression and tRNA modification and aminoacylation. To test this link we spread the *Δsmc* strain on sublethal concentration of mupirocin (an inhibitor of isoleucyl tRNA synthetase [38]). We show that this drug is able to increase 300 times the number of colony forming unit of the *Δsmc* strain at 37°C (Fig 1) indicating clearly that affecting the pool of available charged tRNA could suppress the viability defect of the *Δsmc* strain on rich medium at 37°C.

### Suppressor mutations reduce chromosome segregation and condensation defects caused by the absence of SMC

Suppressor mutations were selected solely for their capacity to restore viability of *Δsmc* mutant in non permissive conditions. We investigated whether they also suppressed or alleviated other defects exhibited by the *Δsmc* mutant cells. Suppressor strains s34 (Δsmc ylbM*), s38 (Δsmc aspS*), and s42 (Δsmc ywlC*) were generally much less sensitive to coumermycin A1 (~500-fold) and to nalidixic acid (~1000-fold, except s34, ~50-fold) than *Δsmc* (S1 Fig). However, when the *ΔylbM* and *ΔywlC* mutations were introduced singly in the wild type background, they sufficed to reduce substantially the sensitivity to both gyrase inhibitors (S2 Fig), indicating that this effect is an indirect effect of the suppressor mutations and not related to the suppression of *Δsmc* defects.

**Fig 2:**
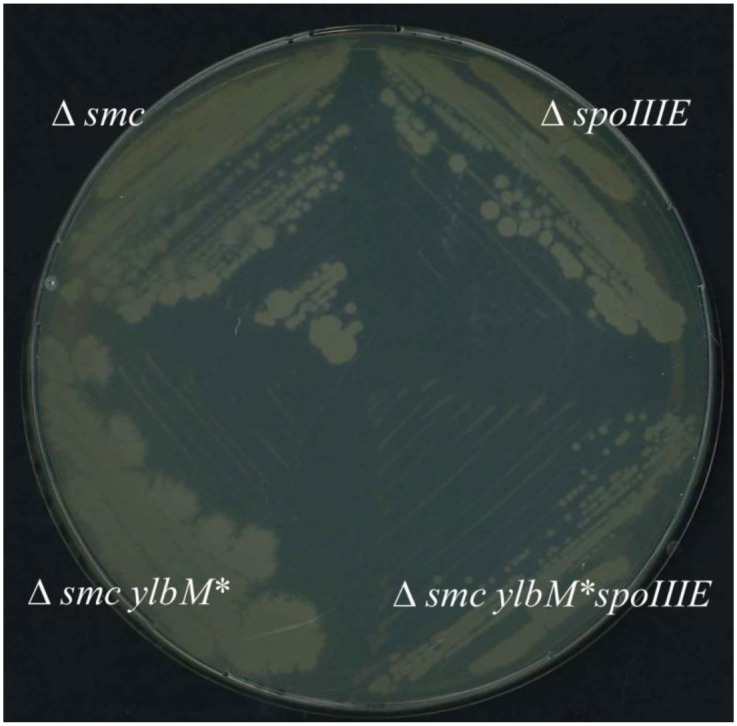
growth of the spoIIIE mutant strains. Δ*smc* cells carrying or not suppressive mutation were combined with the *spoIIIE* deletion. The combination of the two deletion could only be obtain when suppressive mutation is present in the strain. Cells were grown on LB plates incubated at permissive (23°C).

**Fig 3:**
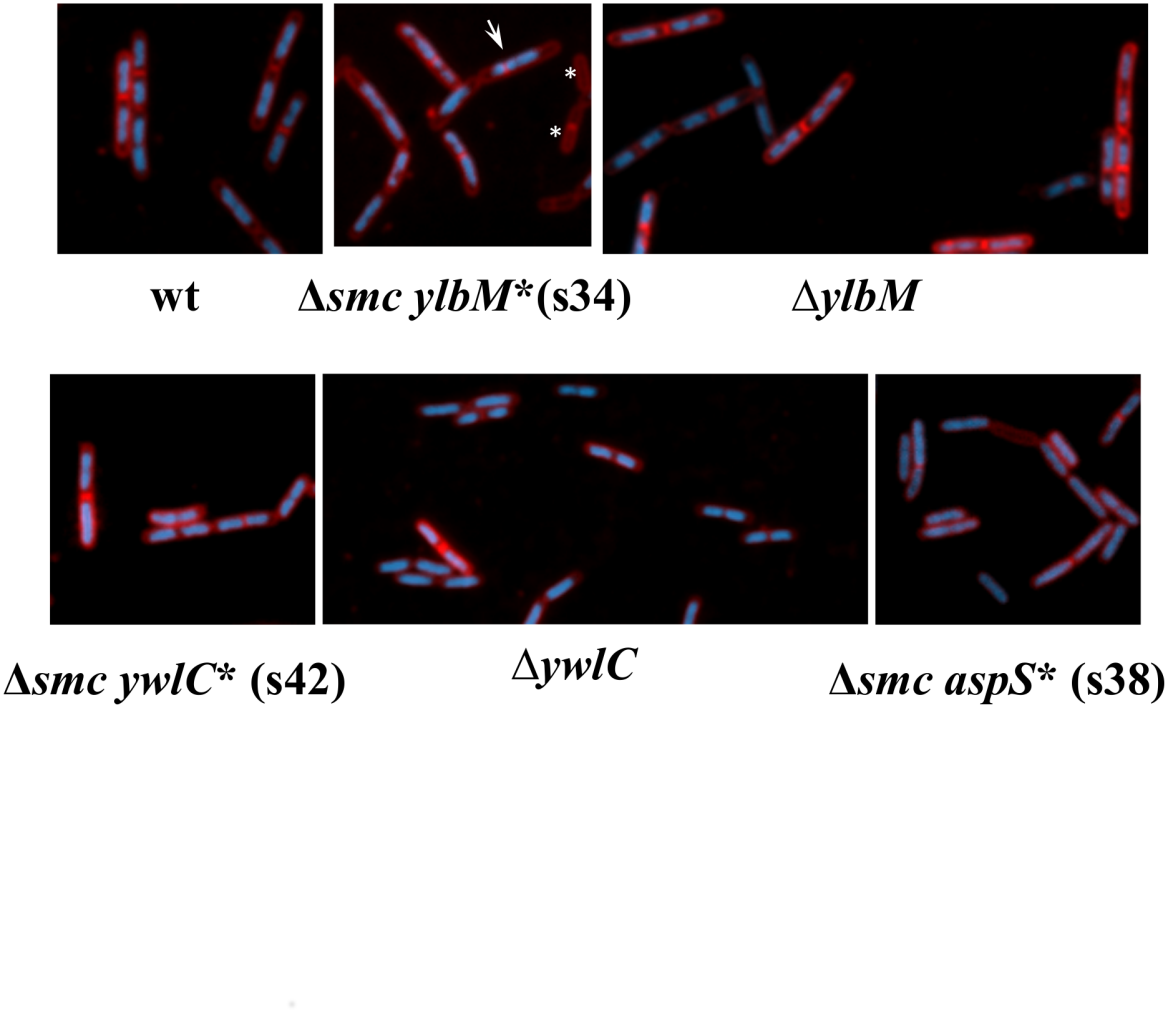
Microscopic observation of suppressors. Cells were grown in LB at 37°C to exponential growth phase (OD_600nm_ between 0.3 and 0.6) and stained with FM4-64FX and DAPI to visualize membranes (red) and DNA (blue) respectively. The white arrow indicates a cell in which division septum was formed on none segregated chromosomes (guillotine phenotype); and anucleate cells are marked with asterisks.

In absence of SMC, the SpoIIIE translocase is essential for cell survival as it rescues missegregated chromosomes trapped in the division septum [39, 40]. To determine whether SpoIIIE is still synthetically lethal with SMC in suppressor strains, we tried to introduce the *ΔspoIIIE* mutation (and *ΔamyE as control)* in the wt, *Δsmc*, s34, 38, and s42 backgrounds. All combinations except the *Δsmc ΔspoIIIE* could be obtained (see Materials and Methods). The resulting strains s34 *ΔspoIIIE*, s38 *ΔspoIIIE*, and s42 *ΔspoIIIE* exhibited slower growth on plates (colonies formed in 48h instead of 24h for suppressor strains on LB at 23°C, Fig 2). These results show that *Δsmc* is not synthetically lethal with *ΔspoIIIE* in a suppressor background, suggesting that segregation defects in *Δsmc* are reduced by suppressor mutations. Supporting this notion, microscopic observation of suppressor strains, grown under permissive conditions, showed that the proportion of anucleate cells was reduced ~2-fold in suppressor strains relative to the *Δsmc* mutant (Fig 3, Table 2). As the single *ΔylbM* and *ΔywlC* mutants exhibited a proportion of anucleate cells similar to wild type, we concluded that suppressor mutations slightly restore chromosome segregation in cells lacking SMC.

### Suppressor mutations induce complex transcriptional changes that overlap the stringent response

To get insight into the global effects of suppressor mutations, we compared the genome-wide transcription profiles of the single *ΔylbM* and *ΔywlC* mutants relative to wild type under one condition (*smc+* background exponential growth in LB at 37°C) that would be non permissive for growth of *Δsmc*. Transcripts were quantified using our recently defined *B*. *subtilis* 168 structural annotation [41] (S3 Table). We found 222 and 1043 genes differentially expressed (FDR < 5%) in *ΔylbM* and *ΔywlC* relative to wild type, reflecting large and complex cellular responses. As changes in mRNA abundance in the *ΔywlC* mutant affected over 18% of the annotated transcripts, the 148 genes differentially expressed in both mutants (S3A Table) likely include genes specific for the suppressor response as well as many non specific genes. When we considered differentially expressed genes in the context of previously defined transcription units (TU) [41], we noted a significant increase of the expression of some genes of the *ilv-leu* operon (start U2191 drive expression of the following TU: *S1073 S1072 S1071 ilvB ilvH ilvC leuA leuB leuC leuD ysoA* S3 Table) encoding enzymes for the synthesis of branched-chain amino acids. A global increase (2 to 8-fold depending on genes) of the expression of the entire operon was observed in both strains with a 5-fold change for *leuABCD* genes. We also observed a significant decrease in the expression of some genes in the *rrn* operon (start U88 drive expression of the following TU: *S49 rplK S50 rplA rplJ rplL ybxB S51 rpoB rpoC S52 rplGB S53 rpsL rpsG fusA S54 tufA ybaC)*, which encodes 7 ribosomal proteins, 2 subunits of the RNA polymerase and 2 translation elongation factors. This decrease affected the whole operon in *ΔywlC* and a similar trend, albeit much weaker, was observed in the *ΔylbM* mutant. Interestingly, both the *ilv-leu* and *rrn* operons were previously reported to be up- and down-regulated, respectively, by the RelA-dependent stringent response to amino acid starvation [42]. This response is induced by a rapid synthesis of (p)ppGpp by RelA, globally causing reallocation of bacterial resources and associated with a decrease of GTP concentration. When the genes previously reported to be under stringent response regulation were mapped to transcription units, we found that 73% (27/37) of these TUs also contained genes differentially expressed in *ΔywlC* (S3B Table). This finding provides a clear signature for an induction of the stringent response within the global transcriptional changes taking place in *ΔywlC*. A similar trend was observed in *ΔylbM* but it was weaker because differential expression of a much smaller number of genes reached statistical significance. We hypothesized that alterations in maturation and amino-acylation of tRNAs caused by the aspS*, ywlC* and potentially ylbM* suppressor mutations could deplete some amino-acylated tRNAs and increase uncharged tRNAs loaded on ribosomes, thereby triggering the stringent response [43]. Different suppressor mutations would constitutively induce the stringent response at various levels and suppress *Δsmc* phenotypes at various degrees.

### Elevated (p)ppGpp dosage in suppressor strains

The stringent response is induced by a rapid synthesis of (p)ppGpp (also known as alarmone) by RelA, the (p)ppGpp synthetase [44]. The increased level of (p)ppGpp causes inhibition of growth and reallocation of bacterial resources [45–47]. Three ppGpp synthetases have been identified in *B*. *subtilis*: a RelA-SpoT homolog (called RelA), which exhibits both the degradation and synthesis activities for (p)ppGpp [48] and two small alarmone synthetases (SAS) called YjbM and YwaC, which have only (p)ppGpp synthetase activity [49].

**Fig 4:**
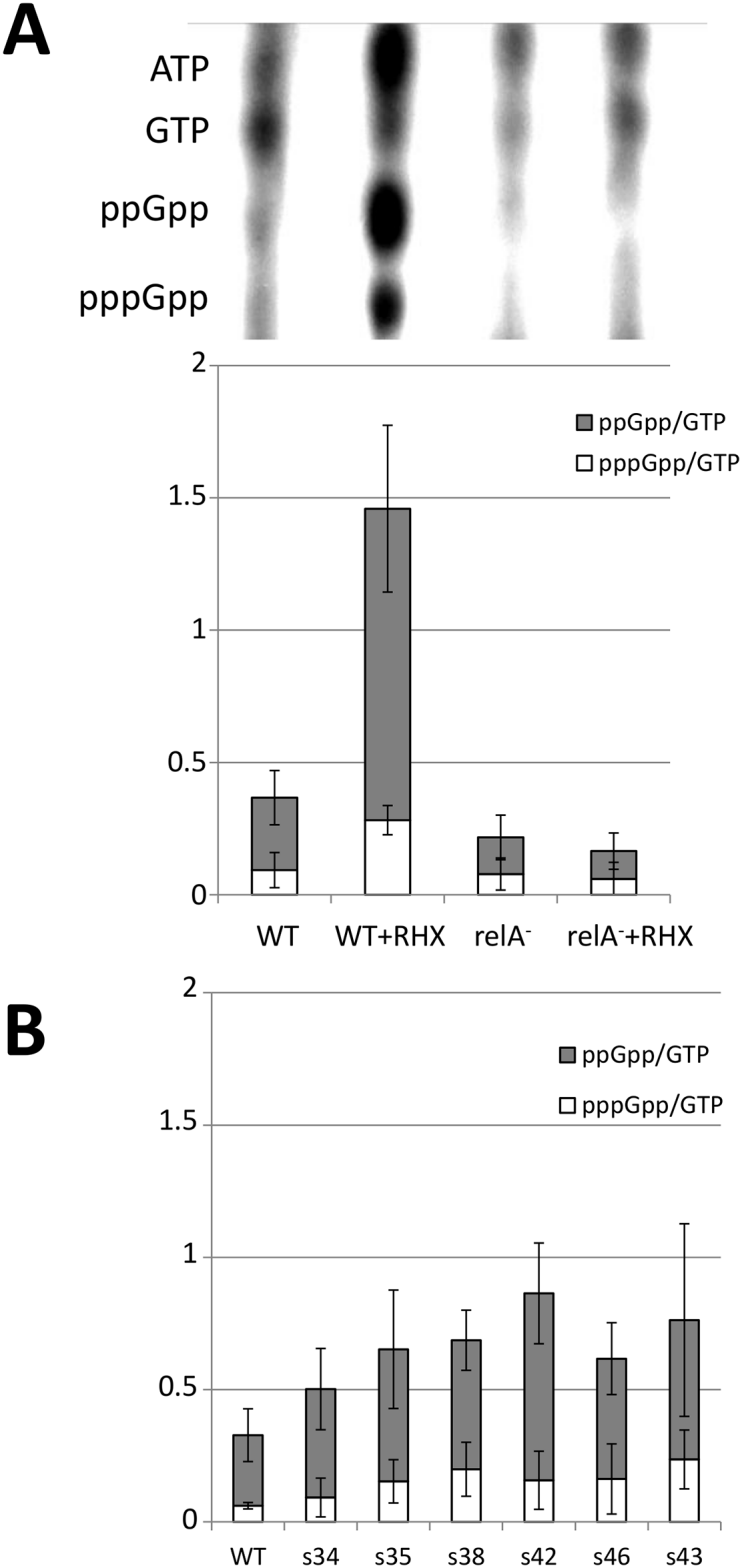
(p)ppGpp quantification. A. Wild type or *relA* mutant strains grown at 37°C in the MOPS derivative medium with or without arginine hydroxamate (100 μg/ml) were labeled with H_3_^32^PO_4_ for 4 to 5 generations. The ^32^P labeled cells were collected and resuspended at the same final concentration in formic acid 1M and frozen. The reaction mixtures were centrifuged for 5 minutes at 8000g and 10μl of supernatant was used for TLC analysis. The position of the signal corresponding to pppGpp, ppGpp, GTP and ATP are indicated. For each strain the ratio of pppGpp/GTP (white) and ppGpp/GTP (grey) were calculated taking the phosphate stoichiometry into account. Each value corresponds to the means and the standard deviation of at least 3 experiments. B. One representative strain of each class of suppressors (with the exception of class VII which is unable to grow in the labeling condition) and the wild type strain were grown, labeled for 4 to 5 generations and the ratio of pppGpp/GTP (white) and ppGpp/GTP (grey) was determined on at least 3 independent experiments.

To determine whether stringent response is induced in suppressor strains, we quantified the cellular pppGpp and ppGpp versus GTP in the different strains, as described in Materials and Methods. As expected the (p)ppGpp/GTP ratio increased 3 to 4 fold when cells were treated with arginine hydroxamate (RHX), a non-functional analog of arginine mimicking arginine deprivation and an inducer of the RelA dependent stringent response (Fig 4A). Similar experiments were performed with one representative strain of each class of suppressors (Fig 4B). The (p)ppGpp/GTP ratio is at least twofold higher in suppressor strains relative to the wild type, except for the s34 (Δsmc ylbM*) strain (~1.5-fold higher). From these experiments, we conclude that the stringent response is induced at various levels in suppressor strains, strongly supporting the notion that this response contributes to the partial restoration of chromosome segregation in *Δsmc* mutant cells.

### Inactivation of the stringent response affects *Δsmc* mutant viability

To determine whether the restoration of the *Δsmc* mutant viability depended on (p)ppGpp accumulation, we constructed *B*. *subtilis* mutants unable to synthetize (p)ppGpp. To this end, the triple mutant’s *Δsmc ΔyjbM ΔywaC* and *ΔrelA ΔyjbM ΔywaC* were constructed by introducing respectively the *Δsmc* and *ΔrelA* mutations in the *ΔyjbM ΔywaC* double mutant (see Materials and Methods). Triple mutants grew on plate and no phenotypic difference in colonies was observed between the *Δsmc* and *Δsmc ΔyjbM ΔywaC* mutants and between the wild type strain and the *ΔrelA ΔyjbM ΔywaC* mutant, as previously reported [49].

**Fig 5:**
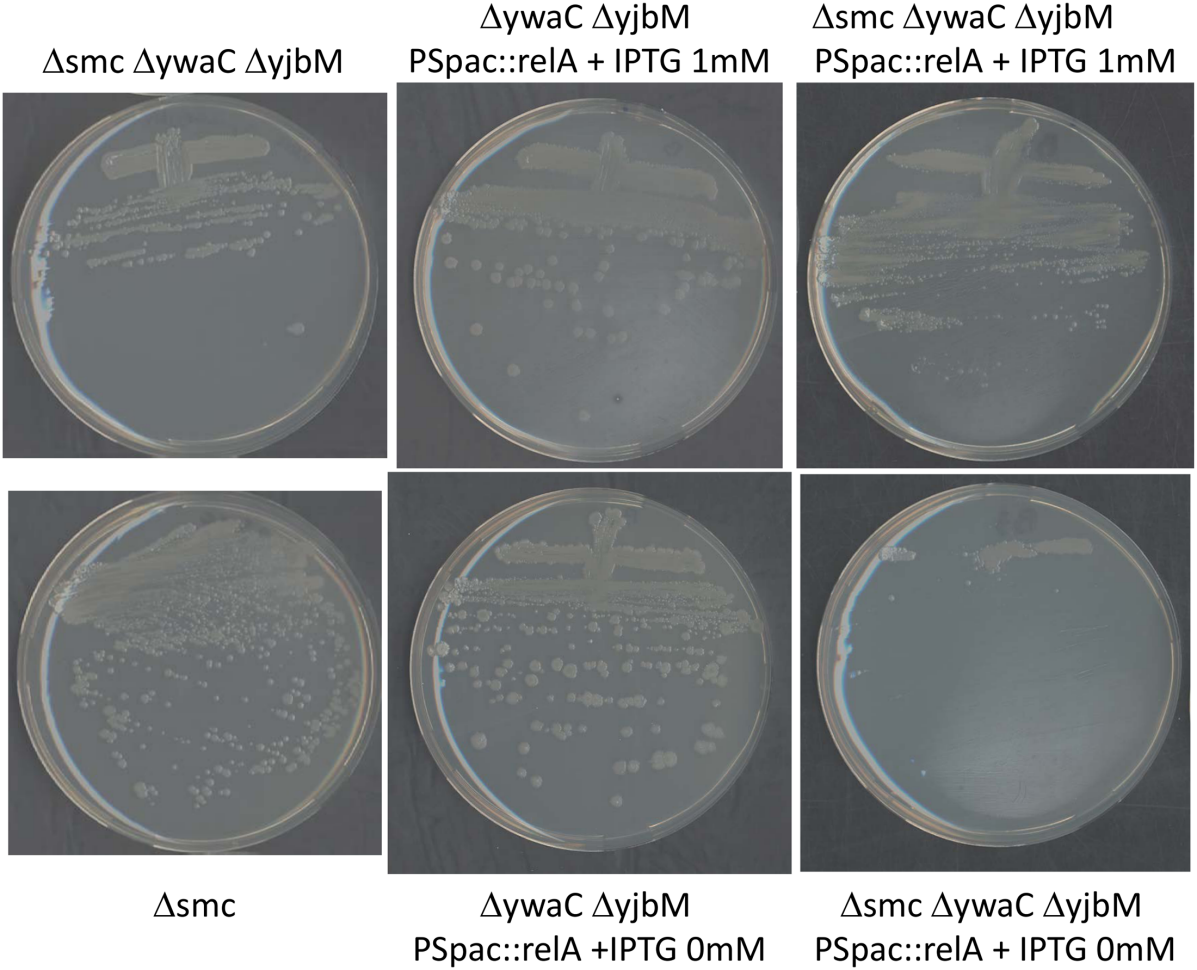
The (p)ppGpp synthase RelA is required for viability of SMC null mutant strains. A strain background was constructed in which the two minor (p)ppGpp synthases were inactivated (*ΔywaC ΔyjbM*) and expression of only remaining (p)ppGpp synthase activity was placed under control of the IPTG-inducible promoter (*PSpac*::*relA*). This strain formed isolated colonies on LB plates without and with IPTG 1mM at permissive temperature (23°C). However, in absence of SMC (Δ*smc*) in this background, IPTG was absolutely required to form isolated colonies. Note that only residual growth in the mass of the streak is observed without IPTG.

The combination of the four deletions was attempted by transforming the triple mutant *Δsmc ΔyjbM ΔywaC* with chromosomal DNA from *ΔrelA ΔyjbM ΔywaC* strain and selecting for the antibiotic resistance marker associated with *ΔrelA* under permissive conditions (LB at 23°C). A total of 30 transformants were obtained from four independent experiments, and their chromosome integrity at the 4 loci was verified (see Materials and Methods). All transformants except one had recovered the wild type *smc* locus. The transformant which carried the *ΔrelA Δsmc ΔyjbM ΔywaC* deletions exhibited a plating efficiency reduced nearly 10-fold relative to *Δsmc* under permissive conditions (S4 Table), suggesting that (p)ppGpp synthesis is important for the fitness of the *Δsmc* mutant. However, since we cannot rule out that the quadruple deletion mutant has acquired compensatory mutation(s), we cannot conclude from these results that (p)ppGpp synthesis is essential for *Δsmc* viability. Thus, we placed the *relA* gene under control of the inducible *pSpac* promoter in the *ΔyjbM ΔywaC* background (see Materials and Methods). The resulting strain did not require IPTG for growth (Fig 5). The *Δsmc allele* was introduced in the presence of IPTG. The resulting strain *Δsmc ΔyjbM ΔywaC pSpac*::*relA* grew similarly to the *Δsmc* and *Δsmc ΔyjbM ΔywaC* strains under permissive conditions (LB + IPTG 1mM at 23°C) (Fig 5). In sharp contrast, when *relA* expression was not induced, the *Δsmc ΔyjbM ΔywaC pSpac*::*relA* strain could not form isolated colonies. Our results strongly support that an active (p)ppGpp synthase is required for the viability of the *Δsmc*.

### Constitutive stringent response restores *smc* null mutant viability

**Fig 6:**
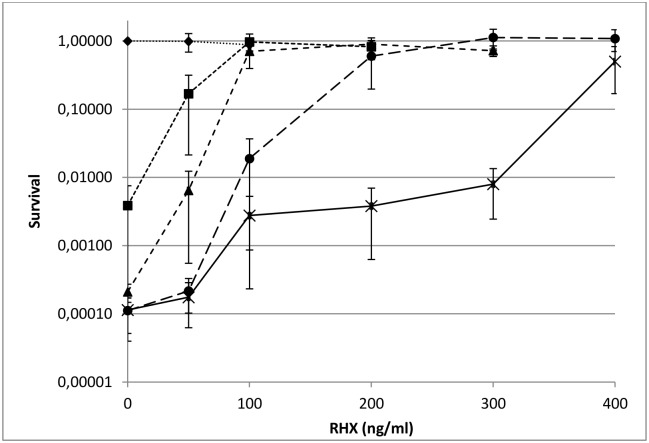
Restoration of Δ*smc* viability using sublethal concentration of RHX. Δ*smc* cells were grown in LB at 23°C to exponential growth phase (OD_600nm_ between 0.3 and 0.6), spread on LB plates supplemented with different concentrations of RHX and then incubated 3 to 4 days at 23°C (diamond), 30°C (square), 37°C (triangle), 42°C (circle) and 51°C (cross). Survival rates were calculated relative to the number of colonies formed on LB at 23°C without RHX. The average and standard deviation of three independent experiments is showed. The value without RHX indicate there is no increase of the viability at high temperature for slow growth Δ*smc* strain. Similar result at 37°C has already been described (17).

If the stringent response suppresses deficiencies in *Δsmc*, accumulation of (p)ppGpp in the suppressor mutants (Fig 4) may be directly linked to the observed suppressor phenotypes. If this hypothesis is correct, artificial induction of the stringent response by sublethal concentrations of aminoacyl tRNA synthetases inhibitors should also restore *Δsmc* viability. *Δsmc* cells were grown under permissive conditions (LB at 23°C), spread on plates containing various concentrations of arginine hydroxamate (RHX), a potent inducer of stringent response, and plates were incubated at different temperatures. Cell survival was plotted as a function of RHX concentration **(** Fig 6). As previously described [17], we observed that sublethal concentrations of RHX could fully restore *Δsmc* viability under all non permissive temperatures up to 51°C. The minimal RHX concentration required for full viability increased with the growth temperature, suggesting that increasing levels of stringent response induction correspond to increasing levels of suppression. Therefore, these data suggest that various levels of induction of the stringent response by RHX suppress *Δsmc* deficiencies under non permissive conditions as efficiently as the suppressor mutations (ylbM* and ywlC*).

### (p)ppGpp synthesis is sufficient for suppression of most defects in *Δsmc* strain

**Fig 7:**
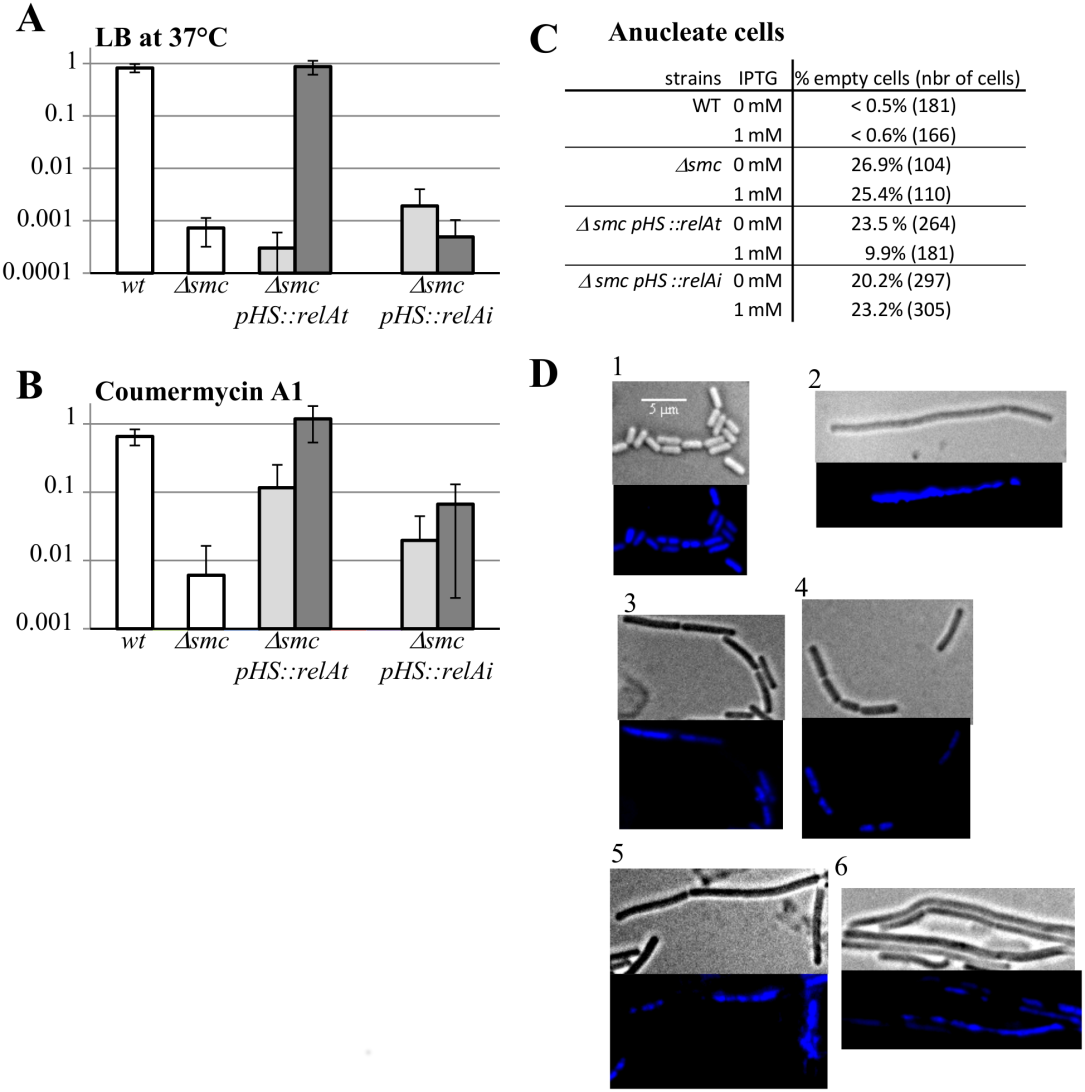
Restoration of Δ*smc* viability by inducible expression of *relA*. *Δsmc* cells carrying different alleles of the *E*. *coli relA* genes either as an inactive form (*relAi*) either as a truncated form (*relAt*) under the transcriptional control of the P_hyperspank_ promoter were grown in permissive condition (LB at 23°C) with or without IPTG 1mM. Cells were then diluted, spread on both LB plates and LB plates containing IPTG and incubated at permissive (23°C) and non permissive temperature (37°C) for 48h or on coumermycinA1 (1μg/ml) at 23°C. For each condition the ratio of cells growing in non permissive condition versus the number of cells grown in permissive condition (panels A and B) was calculated. The results are the means and the standard deviation measured on at least five different clones. Cells grown at 37°C were also analyzed by microscopic observation after DAPI treatment in order to calculate the ratio of anucleate cells (panel C) and to illustrate the condensation phenotype of the DNA in the WT strain (panel D1), *Δsmc* (panel D2), *Δsmc*P_hyperspank_::*relAt* (panel D3), *Δsmc*P_hyperspank_::*relAt* with 1mM of IPTG (panel D4), *Δsmc*P_hyperspank_::*relAi* (panel D5), *Δsmc*P_hyperspank_::*relAi* with 1mM of IPTG (panel D6).

RHX induces the stringent response by mimicking an amino acid starvation, and could have pleiotropic effects unrelated to (p)ppGpp-mediated response. To test whether the viability of *Δsmc* under non permissive conditions could be restored by accumulation of (p)ppGpp independently of amino acid starvation, we used alleles of the *E*. *coli relA* gene: one producing a constitutively active (p)ppGpp synthase (*relAt*) and the other producing a catalytic site mutant inactivated for (p)ppGpp production (*relAi*)[50] (gift from L. Jannière, ISSB, Evry, France). The *relAt* and *relAi* genes were placed under the control of the IPTG-inducible *P*_*HyperSpank*_ *(pHS)* promoter and integrated at the *amyE* locus in the *B*. *subtilis* chromosome. We observed that induction by IPTG of the *relAt* gene resulted in ppGpp accumulation in the cell (ratio (p)ppGpp/GTP increased from 0.45 to 1.14) whereas a similar induction of the *relAi* gene did not ((p)ppGpp/GTP ratios of 0.42 and 0.47 with and without IPTG, respectively).We conclude that the *E*. *coli* RelAt is functional for ppGpp synthesis in *B*. *subtilis*. The strains carrying the *relAt* and *relAi* genes under the control of the IPTG-inducible promoter were grown under permissive conditions (LB at 23°C), diluted 200 times in either LB or LB supplemented with IPTG, incubated for 24h at 23°C and plated on LB with or without IPTG under the various conditions used to test the suppressor phenotypes. Specifically, plating efficiencies relative to the permissive condition (LB at 23°C) were measured for nalidixic acid 2μg/ml, coumermycin A1 0.5μg/ml, and non permissive conditions (LB at 37°C) (Fig 7, S3 Fig). Strikingly, the expression of the constitutively active EcRelAt conferred full viability under non permissive conditions, whereas the inactive EcRelAi did not (Fig 7A). The surviving colonies did not acquire additional chromosomal mutations because they were unable to form colonies when streaked at 37°C without IPTG. Therefore, (p)ppGpp synthesis by EcRelAt suppressed *Δsmc* defects in the absence of amino acid starvation.

The restoration of the other defects exhibited by *Δsmc* was also investigated. The expression of EcRelAt but not of EcRelAi restored *Δsmc* sensitivity to coumermycin to a level similar to wild type (Fig 7B). The uninduced *pHS*::*relAt* provoked a 10-fold increase in the resistance to coumermycin, suggesting a leaky expression from the *pHS* promoter. However, this leakiness was not sufficient to restore *Δsmc* viability under non permissive conditions (Fig 7A). Moreover, the expression of EcRelAt was sufficient to increase the resistance to nalidixic acid (S3 Fig) in a similar range as described for the suppressor strains (Table 1). Cells were observed by fluorescence microscopy after staining of nucleoids with DAPI to determine the proportion of anucleate cells in the population (Fig 7C) and to estimate the chromosome condensation defects of theses strains (Fig 7D). The expression of EcRelAt improved the compaction of the chromosomal DNA and reduced cell filamentation (Fig 7D, compare panels 3 & 4) relative to the *Δsmc* strain (Fig 7D, panel 2), whereas expression of EcRelAi did not (Fig 7D, panels 5, 6). Also, EcRelAt expression reduced by ~2-fold the proportion of anucleate cells (Fig 7C), indicating an improvement of chromosome segregation.

Altogether, our data indicate that all the phenotypes observed in suppressor strains were reproduced by expressing a constitutive (p)ppGpp synthase *EcRelAt* in the cell. Importantly, whereas (p)ppGpp synthesis fully restored viability of *Δsmc* cells under non permissive conditions, it only partially restored nucleoid compaction and segregation in *Δsmc* cells.

## Discussion

We report the identification and the characterization of suppressor mutations able to restore viability of the *Δsmc* mutant under non permissive conditions. Three suppressor mutations partially or completely inactivated genes involved (*aspS* and *ywlC*) and potentially involved (*ylbM*) in tRNA modification and aminoacylation. These suppressor mutations corrected or alleviated most defects exhibited by cells lacking SMC. Genetic analysis indicated that the stringent response induced in these suppressor strains was essential to restore *Δsmc* mutant viability under non-permissive conditions (this confirmed already published observation [17]). We observed the inability to inactivate (p)ppGpp synthetases in the *Δsmc* mutant without causing loss of cell viability even under permissive conditions. This finding together with the rescue of *Δsmc* viability by production of an active (p)ppGpp synthase in the cell indicated that (p)ppGpp plays a crucial role for cell viability in absence of SMC. We found that induction of the stringent response reduced sensitivity of *Δsmc* to the gyrase inhibitor coumermycin A1 and partially alleviated its defects in chromosome compaction and segregation. These observations indicate that complete cell viability can be restored even when segregation defects persist, suggesting that a normal segregation cannot be restored in the absence of the SMC-ScpAB complex.

Is the stringent response alone responsible for the restoration of *Δsmc* viability in suppressors? We found that the level of accumulation of ppGpp in the cells, measured by the (p)ppGpp/GTP ratio, was lower in suppressor mutants than in cells treated by RHX, indicating that stringent response is only partially induced in suppressors. Moreover, no clear correlation between the (p)ppGpp/GTP ratio in suppressors and their phenotypes could be observed (comparing (p)ppGpp levels with the coumermycin A1 sensitivity, proportion of anucleate cells and growth rate (S1 Fig, also estimated by the colony size, Table 1). Thus, even though induction of stringent response takes place in every suppressor tested, a part of suppressors phenotypes could be specific of the suppressor allele and not directly linked to (p)ppGpp level.

How can the stringent response restore viability in absence of SMC?

The effector molecule (p)ppGpp exerts a global impact on bacterial physiology and thereby affects many cellular processes including transcription, translation, and DNA replication (for review see [51]). An attractive hypothesis is that SMC-ScpAB has an essential role in coordinating chromosome segregation with the other cellular processes affected by the stringent response. However, to determine precisely how the stringent response could restore deficiencies caused by lack of SMC-ScpAB, we would need to be able to separate the effects of (p)ppGpp on each process, for example on rRNA transcription, genome integrity and/or DNA replication. This currently represents a challenge difficult to tackle experimentally.

One effect of the stringent response is to slow down or stop DNA replication through (p)ppGpp-mediated inhibition of the DNA primase DnaG, an enzyme essential for lagging strand synthesis and initiation of replication [29, 52]. Gruber *et al*. [17] propose that slowing down DNA replication by stringent response would give more time for the separation and segregation of newly synthesized chromosomes in absence of the SMC-ScpAB complex. Fast moving replication forks would require SMC-ScpAB to support the timely organization and segregation of region near origin of replication. In support of this notion, we observed as Gruber *et al*.[17] that *s*ub lethal concentrations of hydroxyurea (HU), a drug slowing down DNA replication forks by specifically depleting the cell dNTP pools through inhibition of class I ribonucleotide reductase (RNR) [53–55], also partially restored *Δsmc* viability at 30°C on LB (data not shown). Because HU may have a wide range of additional effects, including affecting the NTP pools and triggering the stringent response, the interpretation of such results remains difficult.

Taking in account the localization of the suppressive mutations in genes involve in the transcription/translation process we propose an alternative but not exclusive hypothesis. The stringent response in *B*. *subtilis* affects a large set of genes [42], and especially causes a strong decrease in *rrnO* and *rrnJ* expression, two major rRNA operons in *B*. *subtilis* [28]. To date it is not known whether the SMC-ScpAB complexes in bacteria play any biological role in the integrity and dynamic of highly transcribed regions like rRNA operons. However, it is intriguing that rRNA operons are bound preferentially by SMC complexes [25]. Moreover, in *E*. *coli*, DksA was isolated as a multicopy suppressor to the temperature-sensitive colony formation of *muk* mutant [56]. DksA is necessary to trigger the stringent response and was described to potentiate (p)ppGpp regulation, notably inhibition of rRNA promoters and activation of amino acid promoters [57, 58]. In addition, DksA can inhibit the stringently repressed promoter *rrnB*-P1 independently of (p)ppGpp, and the overproduction of DksA can completely compensate most of the defects presented by cells unable to produce (p)ppGpp [59, 60]. Thus, it appears in *E*. *coli* that inhibition of rRNA operons might be responsible for the suppression of *mukB* mutant. Furthermore, in budding yeast, the condensin complex is required for a correct segregation of rRNA coding region [61–64]. Hence, DNA regions encoding rRNA operons, which are subjected to high levels of transcription and particular DNA topological constraints, would need a specific machinery to assist their separation after replication. Similarly, the *B*. *subtilis* SMC-ScpAB complex could maintain the integrity of the highly transcribed regions and assist their proper segregation. In absence of SMC-ScpAB, the RNA operon could be difficult to be segregate; as some of these operons are close to the origin of replication the defect could be described as a default in the origin region segregation. The reduction of rRNA operons transcription by stringent response (also observed in the *ΔywlC* mutant strain) would become essential to enable correct partitioning of these regions, thereby restoring viability of cells lacking SMC-ScpAB.

Finally, the SMC-ScpAB complex has been extensively studied and a mechanistic model of “condensation centers” has emerged, in which the chromosome compaction activity is localized and synchronized with the cell cycle [15, 24, 25]. Our results lead us to propose that suppression of the lack of SMC by the stringent response could be due to an effect on the reduction of the level of expression of rRNA operons (and maybe also a reduction of the speed of replication). As counterpart of the “condensation center” model, the need to coordinate efficiently segregation, transcription with replication would be less critical in absence of DNA region particularly difficult to segregate especially if replication proceeds slowly. Consistent with this notion, bacterial species that have lost bacterial condensin without major impact on their viability under standard growth conditions (including *Pseudomonas aeruginosa*, *Staphyloccus aureus*, *Deinococcus radiodurans and Mycobacterium tuberculosis)* exhibit longer generation times compared to the rapidly dividing *E*. *coli* and *B*. *subtilis* [9, 10, 26, 65, 66].

## Materials and Methods

### Bacterial strains, and growth media

*Bacillus subtilis* strains used is this study are listed in Table 3. Cells were grown in Luria Bertani (LB) rich medium, or minimal medium salts (MM) supplemented with trace elements solution [67] and the appropriate amino acids.

**Table 3:** Strains used in this study.

Strain	relevant genotype	reference
168	*trp*C2	laboratory stock
EDJ180	168 *smc*::*specR*	26
EDJ547	168 *pMUTIN*::*addB*	Benoist, in preparation
EDJ660	168 *AmyE*::*Psweet*	Benoist, in preparation
EDJ672	168 *recU*::*CmR*	Benoist, in preparation
CB 182	168 *ylbM*::*CmR amyE*::*Pxyl*-*ylbM-SPA*	This work
CB 139	168 *spoIIIE*::*CmR*	This work
EDJ834	168 *smc*::specR ylbM*(Δ232–264)	This work
EDJ838	168 *smc*::specR aspS* (V_432_>G)	This work
EDJ842	168 *smc*::specR ywlC* (P_64_>L)	This work
CB 330	168 *Δupp*::*λPr-neo smc*::*specR* Δ *ywaC yjbM*:: *upp-phleo-cI*	This work
CB 329	168 *Δupp*::*λPr-neo relA*::*eryR* Δ *ywaC yjbM*::*upp-phleo-cI*	This work
EDJ 983	168 *Δupp*::*λPr-neo smc*::*specR relA*::*eryR yjbM ywaC*::upp-phleo-cI mut*	This work
CB 156	*Δupp*::*λPr-neo*, ylbM* (Δ 232–264)	This work
CB 160	*Δupp*::*λPr-neo*, ywlC* (P_64_>L)	This work
CB 167	168 *ylbM*::*CmR*	This work
CB 169	168 Δ *ywlC*::*CmR*	This work
CB 171	168 *smc*::*specR ylbM*::*CmR*	This work
CB 173	168 *smc*::*specR ywlC*::*CmR*	This work
CB 331	168 *smc*::specR aspS* (V_432_>G) pDG148-*aspS*	This work
EDJ1092	168 *smc*::*specR (pHyperspank*::*relAt)CmR*	This work
EDJ1096	168 *smc*::*specR (pHyperspank*::*relAi)CmR*	This work
EDJ1164	168 *pSpac*::*relA*::*EryR*	This work
EDJ1166	168 *Δupp*::*λPr-neo* Δ*ywaC yjbM*:: *upp-phleoR-cI pSpac*::*relA*::*EryR*	This work
EDJ1168	168 *Δupp*::*λPr-neo* Δ*ywaC yjbM*:: *upp-phleoR-cI pSpac*::*relA*::*EryR smc*::*specR*	This work

Total DNA was extracted from *B*. *subtilis* as described previously [68]. Competent cells and transformation were done as previously described [69].

Antibiotics were used at the following concentrations: chloramphenicol 6 μg/ml, phleomycin 2 μg/ml, erythromycin 0.3 μg/ml, kanamycin 6 μg/ml, spectinomycin 50 μg/ml, tetracyclin 7.5 μg/ml. For the restoration of the viability of *smc* null mutant, concentrations of arginine hydroxamate (RHX) used are specified. *E*. *coli* TG1 cells were grown in LB medium supplemented with 100 μg/ml of ampicillin for plasmid maintenance; plasmids were extracted using the alkaline lysis method [70].

### Oligonucleotides, PCR amplification and DNA sequencing

Oligonucleotides were purchased from MWG-Biotech (Germany). The list and sequence of the oligonucleotides used in this study are available upon request. PCR amplifications were performed as recommended by the enzyme supplier (Takara), except when specified. DNA sequencing was performed on PCR products treated with exonuclease I and shrimp Alkaline phosphatase (Amersham), using the Applied Biosystems PRISM BigDye terminator sequencing kit, a Perkin Elmer 9600 thermal cycler, and an Applied Biosystems 3700 DNA analyzer.

### Construction of *B*. *subtilis* strains

-For deletion of *relA*, *spoIIIE*, *ylbM* and *ywlC*, either the erythromycin-resistance gene (*eryR*) or chloramphenicol-resistance gene (*CmR*) were amplified respectively from pMUTIN4 [71] or pUC19-*uppphleoR* [72] plasmids as templates. For each gene to be deleted, the PCR primers used were designed to amplify flanking regions corresponding to 1kb to 1.5kb upstream and downstream of the target genes. The primers intended to immediately flank the antibiotic cassette were also designed to incorporate a 21 to 24 bp terminal homologous to the antibiotic resistance gene DNA fragment. For each deletion, three PCR products corresponding to the upstream region, the downstream region, and the antibiotic resistance cassette replacing the targeted gene were mixed in equimolecular amounts, and joined using a subsequent PCR reaction. The resulting DNA fragment was used to transform *B*. *subtilis* 168 competent cells and transformants were selected for the appropriate incoming antibiotic resistance. The proper integration and the loss of the wild type gene were verified by PCR. In addition, the sequence of the PCR amplified region was verified.

Introduction of ylbM*(Δ232–264) or ywlC* (P_64_>L) mutations and deletions of *ywaC* or *yjbM* genes in wild type strain were carried out according the pop-in pop-out system [72, 73].

For pDG148-*ylbM*, pDG148-*ywlC* and pDG148-*aspS*; the regions from the RBS to the stop codon of *ylbM*, *ywlC* and *aspS* genes respectively were amplified using primers either with extensions carrying HindIII and SalI restriction sites for *ylbM* and *ywlC* or SalI and SphI for *aspS* at the 5’ and 3’ ends respectively. PCR products were consequently cloned into HindIII and SalI digested pDG148.

The CB182 strain expressing the YlbM-SPA fusion protein was constructed as follows. The *ylbM* ORF was amplified using primers adding AvrII and NcoI restrictions sites at the 5’ and 3’ ends respectively and was cloned between the same restriction sites of the pFL40 plasmid [74] in fusion with the SPA tag. The plasmid was first constructed and verified in *E*. *coli* before being transformed to competent CB 167 cells.

To test the functionality of YlbM-SPA, *Δsmc ΔylbM* DNA was used to transform *ΔylbM amyE*::*Pxyl*:*ylbM-SPA* competent cells. Plating efficiency of the resulting *Δsmc ΔylbM amyE*::*Pxyl*:*ylbM-SPA* strain on LB supplemented with D-xylose was 10^−4^ lower at 37°C than at 23°C (data not shown) indicating that YlbM-SPA protein is functional.

The expression of *relA gene* under the control of the IPTG-inducible promoter *PSpac* was achieved by PCR amplification of the 5’ gene region (~500 bp) including the putative ribosome binding site (RBS), cloning of the fragment into pMUTIN4 vector, and integration of the resulting vectors into the chromosome by single-crossover, as previously described[71]. The correct integration was verified by PCR and sequencing.

An integration vector (gift from L. Janniere, ISSB Evry, France, to be published elsewhere) was used to insert by transformation the *relAi* and *relAt* genes [50] under the HyperSpank promoter at the *amyE* locus of the wild type (168) strain and the *Δsmc* isogenic strain.

### Microscopy experiments

Strains, grown under appropriate conditions as described in the text, were stained with FM4-64FX (Molecular Probes) to visualize the cell membrane and with 4’,6-diamino-2-phenylindole (DAPI, Molecular Probes) to visualize the nucleoid. Cells were deposited on slides covered with 1.2% agarose in minimal medium [75]. Cells were examined using a Leica DMRA2 microscope and a COOLSNAP camera (Roper Scientific USA). Images were captured and analyzed using Metamorph V6.3r5.

### Inactivation of SpoIIIE in suppressor strains

Suppressors were transformed with chromosomal DNA carrying the deletion of *spoIIIE*, and the wild type locus of *smc*. Transformants were selected for incoming marker at 23°C (*spoIIIE* deletion) on rich medium. In order to estimate the ability to inactivate this gene in suppressor, the transformants were tested for their ability to carry both of the two antibiotic resistance genes (ie marker of *smc* deletion and *spoIIIE* deletion marker). If more than 50% of the transformants have the two markers then the strain could be constructed. In that case two transformants have been tested by PCR to verify the chromosomal organization at the vicinity of the two loci.

### Purification of YlbM-SPA fusion from *B*. *subtilis*

Overnight cultures grown in LB medium with erythromycin and IPTG (1mM) at 37°C were diluted 100 fold in 2 liters of the same medium with 0.5% D-xylose. The cultures were incubated with aeration at 30°C until growth reached OD_600nm_ ~ 0.5. Cells were harvested, washed in buffer A (10 mM Tris-HCl pH 7.5, 150 mM NaCl), frozen in liquid nitrogen and stored at –80°C. YlbM-SPA was purified as previously described [74]. Proteins complexes were analyzed by 12.5% SDS-PAGE followed by Coomassie blue staining (Bio-Safe Coomassie, BIORAD) and interacting proteins are identified by LC-MSMS mass spectrometry as previously described [76].

### Transcriptomic analyses

Δ*ywlC* and Δ*ylbM* strains grown in LB medium were harvested in exponential phase (O.D._600nm_ around 0.5) RNA was extracted following the method described by Nicolas [41]. The RNA concentration was measured using Nanodrop and RNA quality was determined using an Agilent 2100 bioanalyser. Labelling and hybridization of the sample were performed under the conditions recommended by NimbleGen and as previously described [77]. Transcriptomic results were analysed [41]. An aggregated expression value was computed for the genes annotated in the GenBank file AL009126.3 and the newly defined transcribed regions. Gene expression values were quantile-normalized between experiments. Differential expression analysis was performed using a negative binomial (NB) regression model. The dispersion parameter of the NB model was estimated by mean-dependent local regression using DESeq, which was shown to be appropriate for small numbers of biological replicates, In addition to the log2 fold change, we reported the minimum false discovery rate and the pvalue corresponding to each effect for each gene to capture the effect of both *ΔylbM* and *ΔywlC* mutant compare to the wild-type (WT). The functional annotation was extracted from SubtiWiki database (http://subtiwiki.uni-goettingen.de/).

### Suppressors sequencing

Next Generation Sequencing (NGS) was performed on a SOLiD v3.5 machine. Fragment libraries were prepared according to the published protocol (Applied Biosystems). Libraries were barcoded to allow for multiplexing of samples. 35-base reads were generated and 11 to 13 million tags were generated for each sample. These reads were mapped to the *B*. *subtilis* reference sequence (4215606bp) (strain 168, EMBL/GenBank/DDBJ entry AL009126.3). The software CORONA (Applied Biosystems) was used for mapping tags to the reference sequence, permitting up to 3 mismatches. These criteria allowed matching of approximately 60% of tags. SNP detection was also performed on the SOLiD cluster using the CORONA software with its default settings.

### Test effect of identified mutations

We developed two approaches to further characterize the effect of these mutations:

1. for non-essential genes carrying a potential suppressor mutations, we first introduced each mutation individually in wild type strain, then the *smc* gene was deleted under permissive conditions. Finally we tested the plating efficiency at 37°C on rich medium.
2. for essential genes carrying potential suppressor mutations, suppressor strains were transformed using the replicative plasmid pDG148 derivatives carrying the wild type copy of the mutated chromosomal gene under the control of an IPTG inducible promoter Pspac, and then we measured viability of such strain on rich medium at 37°C with and without IPTG.

### ppGpp quantification

For (p)ppGpp dosage cells were grown in modified phosphate starvation medium [50 mM morpholinopropanesulfonate (adjusted to pH 7.0 with KOH), 0.4 mM KH_2_PO_4_, 30.27 mM (NH_4_)2SO_4_, 6.88 mM Na-citrate, 0.2% glucose, 0.1% of casamino acids,0.01% tryptophan 3.5 mM MgSO_4_, 0.01 mM ZnSO_4_, 28mM MnSO_4_, 0.304 mM FeCl_3_,]. To label *B*. *subtilis* previously published protocol [29] was adapted: exponentially growing cells were diluted in the same medium with 30 mCi ml^-1 32^P orthophosphate (900 mCi mmol^-1^, Perkin Elmer) for 4–5 generations. Cells were concentrated by centrifugation (1 minute at full speed), then nucleotides were extracted in 1 M formic acid and loaded on PEI cellulose plates (JT. Baker). Plates were developed in 1.5 M KH_2_PO_4_ (pH 3.4), exposed onto a Storage Phosphor Screen, and scanned using a GE Storm Scanner. Spots were quantified using ImageQuant software. All nucleotides were normalized by phosphate number and expressed as molar ratios to GTP.

## Supporting Information

S1 Fig Gyrase inhibitor sensibility on LB at 23°C. (PDF)

S2 Fig Gyrase inhibitor sensibility on LB of the Δ*smc*, Δ*ylbM*, and Δ*ywlC* mutants. (PDF)

S3 Fig Induction of a nalidixic acid resitance phenotype by inducible expression of two alleles of *relA*. (PDF)

S1 Table List of mutated genes identified by SoliD sequencing of one representative strain of each suppressor class. (PDF)

S2 Table YlbM-SPA partnership obtained by tandem affinity purification (TAP). (PDF)

S3 Table A-Expression levels in strains carrying single suppressor mutation. B- Distribution of differentially expressed genes in transcription units known to be regulated by stringent response. (XLSX)

S4 Table Comparison of the number of colonies formed per OD_600nm_ unit. (PDF)

## Abbreviations

SMC: Structural Maintenance of Chromosomes
MC: Mitomycin C
RHX: arginine hydroxamate
