# Supplementary material for: Constitutive Stringent Response Restores Viability of Bacillus subtilis Lacking Structural Maintenance of Chromosome Protein
Source: PLoS One. 2015 Nov 5;10(11):e0142308. doi: 10.1371/journal.pone.0142308 (PMC4634966; doi:10.1371/journal.pone.0142308)
Supplement: S1 Fig — (PDF) [file pone.0142308.s001.pdf]

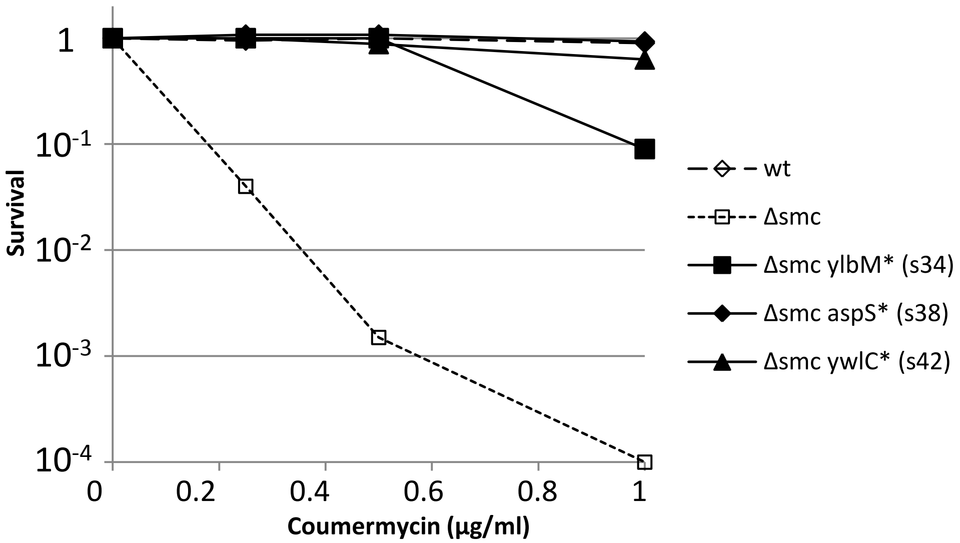

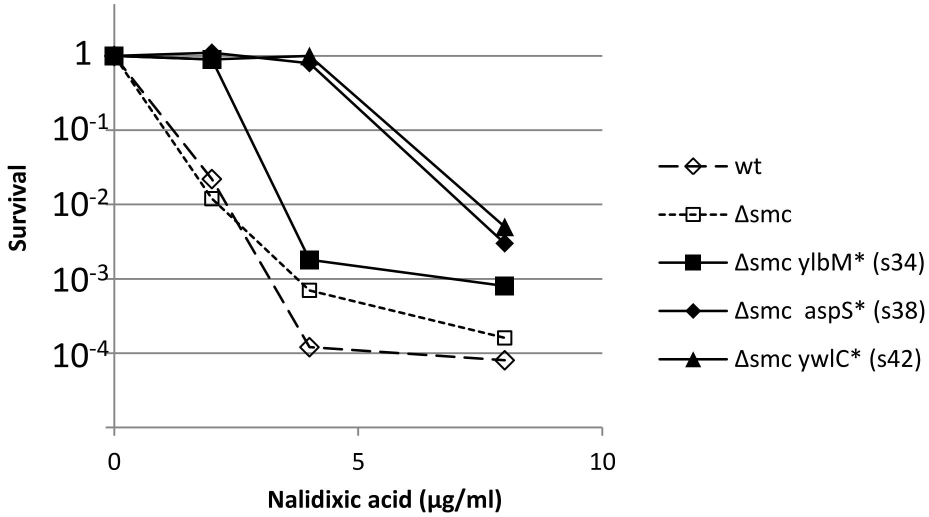

S1 fig.: Gyrase inhibitor sensibility on LB at 23°C

Cells were grown in rich medium at 23°C to exponential grow phase (OD_{600nm} between 0.3 and 0.6), and spread on LB plate supplemented with different concentrations of coumermycin A1 (upper panel) or nalidixic acid (bottom panel) and incubated 4 days at 23°C. The survival of wild type (empty diamonds), Δ*smc* (empty squares), Δ*smc* ylbM* (open squares), Δ*smc* aspS* (open diamonds) and Δ*smc* ywlC* (open triangles) to coumermycin A1 (upper panel) and to nalidixic acid (bottom panel) were calculated by comparison between the number of viable cells with and without drug. Experiments were done three times and one representative result is shown.
