# Supplementary material for: Constitutive Stringent Response Restores Viability of Bacillus subtilis Lacking Structural Maintenance of Chromosome Protein
Source: PLoS One. 2015 Nov 5;10(11):e0142308. doi: 10.1371/journal.pone.0142308 (PMC4634966; doi:10.1371/journal.pone.0142308)
Supplement: S2 Fig — (PDF) [file pone.0142308.s002.pdf]

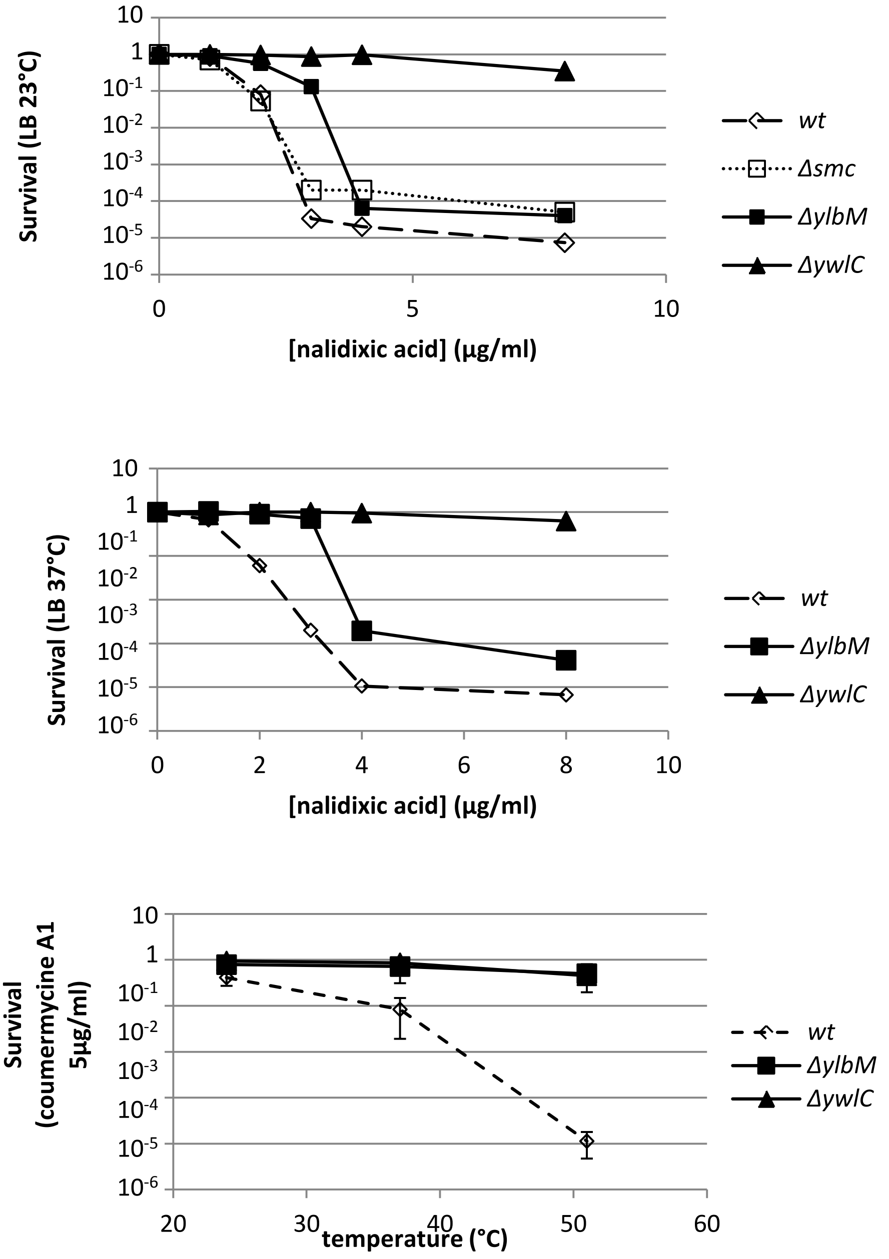

S2 Fig. : **Gyrase inhibitor sensibility on LB of the** Δ*smc*, Δ*ylbM*, and Δ*ywlC* **mutants**
Cells were grown in rich medium at 23°C to exponential grow phase (OD_{600nm} between 0.3 and 0.6), and spread on LB plate supplemented with different concentrations of coumermycin A1 or nalidixic acid and incubated at 23°C. At 51°C, nalidixic acid did not show any effect on viability regardless of the concentration used, suggesting that nalidic acid could be degraded or inactive at this temperature. Each panels shows survival of wild type (empty diamonds), Δ*smc* (empty squares), Δ*ylbM* (open squares), and Δ*ywlC* (open triangles) after exposure to nalidixic acid (upper panel) and to coumermycin A1 (bottom panel), as indicated. Experiments were done three times and one representative result is shown.
