# Supplementary material for: Constitutive Stringent Response Restores Viability of Bacillus subtilis Lacking Structural Maintenance of Chromosome Protein
Source: PLoS One. 2015 Nov 5;10(11):e0142308. doi: 10.1371/journal.pone.0142308 (PMC4634966; doi:10.1371/journal.pone.0142308)
Supplement: S3 Fig — (PDF) [file pone.0142308.s003.pdf]

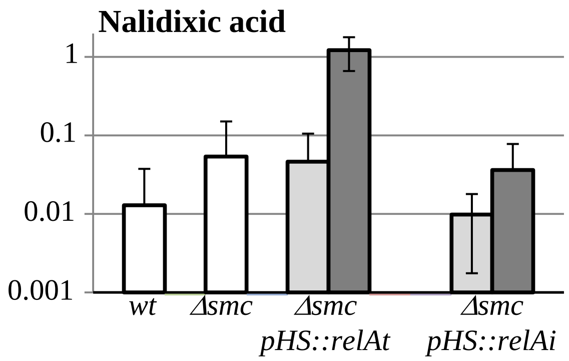

S3 Fig. : Induction of a nalidixic acid resitance phenotype by inducible expression of two alleles of *relA*
Δ*smc* cells carrying different alleles of the *E.coli relA* genes either an inactive form (*relAi*) or a truncated form (*relAt*) under the transcriptional control of the $P_{hyperspank}$ promoter were grown in permissive condition (LB at 23°C) (colored light grey) or with IPTG 1mM (colored dark grey). Cells were then diluted, spread on LB plates in the presence and absence of IPTG and incubated at permissive (23°C) and on LB plates with nalidixic acid (1µg/ml) at 23°C. We calculated for each condition the ratio of cells growing in non permissive condition versus the number of cells grown in permissive condition.
