# Supplementary material for: Constitutive Stringent Response Restores Viability of Bacillus subtilis Lacking Structural Maintenance of Chromosome Protein
Source: PLoS One. 2015 Nov 5;10(11):e0142308. doi: 10.1371/journal.pone.0142308 (PMC4634966; doi:10.1371/journal.pone.0142308)
Supplement: S1 Table — (PDF) [file pone.0142308.s004.pdf]

Strains	Coverage	Mutations localization
**s34 (class I)**	194	***ylbM***
**s35 (class II)**	82	*ybaR*<br>*ansZ*<br>*citR*<br>*ykuI* (inter) *ykuJ*<br>*ypmT*<br>*trpC*<br>*nadD*<br>*lonB*<br>*sigY*<br>*tetB*<br>*exoA* (inter) *rpsR*
**s38 (class III)**	287	***aspS***
**s46 (class IV)**	198	No mutation validated
**s43 (class V)**	60	No mutation validated
**s42 (class VI)**	68	***ywlC***
**s33 (class VII)**	28	*yceD*<br>*ygaN*<br>*yhaJ*<br>*pbpF*<br>*yhjP*<br>*kdgR*<br>*aspB*<br>*ccA*<br>*nadR*<br>*bioI*<br>*pbpD*<br>*mrpD*<br>*yumB*<br>*pucG*<br>*liaS*<br>*hisF*<br>*swrAA*<br>*ywiE* (inter) *narI*<br>*scoA*<br>*wapA*

Table S1: List of mutated genes identified by SoliD sequencing of one representative strain of each suppressor class.

This table shows results of high throughput sequencing of genome of one representative strain of each suppressor classes. Coverage indicated the total length of sequence, given in genome

equivalent, which have been obtained for each of the sequenced suppressor strain. SNP detected by CORONA (Applied Biosystems) software: (i) no mutation validated indicated no mutation detected after the CORONA analysis (ii) all the mutations detected after CORONA analysis and (iii) in bold mutation detected by CORONA analysis, verified by Sanger sequencing and responsible for the suppression phenotype (see the main text for the validation). Mutations localized between two genes are indicated by (inter).
