# Supplementary material for: Constitutive Stringent Response Restores Viability of Bacillus subtilis Lacking Structural Maintenance of Chromosome Protein
Source: PLoS One. 2015 Nov 5;10(11):e0142308. doi: 10.1371/journal.pone.0142308 (PMC4634966; doi:10.1371/journal.pone.0142308)
Supplement: S2 Table — (PDF) [file pone.0142308.s005.pdf]

Protein	Function (associated processes)	PAI
YlbM	unknown	44.7
Pgm	Phosphoglyceromutase (glycolysis / gluconeogenesis)	0.74
CcpA	LacI family transcription regulator (mediates carbon catabolite repression)	0.46
AnsB	aspartate ammonia-lyase (aspartate degradation)	0.38
GltX	glutamyl-tRNA synthetase (translation)	0.28
CodY	transcriptional repressor CodY (regulation of a large regulon in response to branched-chain amino acid limitation)	0.27
Pta	phosphotransacetylase (overflow metabolism)	0.23
DnaN	DNA polymerase III subunit beta (DNA replication. DNA repair)	0.21
Pgi	glucose-6-phosphate isomerase (glycolysis / gluconeogenesis)	0.21
BipA	homolog of GTPase (probably involved in ribosome assembly)	0.18
DapA	dihydrodipicolinate synthase (lysine and peptidoglycan biosynthesis)	0.17
CitG	fumarate hydratase (TCA cycle)	0.17
FtsZ	cell division protein FtsZ (formation of Z-ring)	0.15
GndA/YqjI	6-phosphogluconate dehydrogenase (pentose phosphate pathway)	0.15
PheT	phenylalanyl-tRNA synthetase subunit beta (translation)	0.15
GlyS	glycyl-tRNA synthetase subunit beta (translation)	0.13
ValS	valyl-tRNA synthetase (translation)	0.11
ArgS	arginyl-tRNA synthetase (translation)	0.09
MetS	methionyl-tRNA synthetase(translation)	0.09
WapA	cell wall-associated protein (unknown)	0.02

Table S2. YlbM-SPA partnership obtained by tandem affinity purification (TAP)

After purification of YlbM-SPA as described in Materials and Methods the eluted proteins were separated on a 12.5% SDS-PAGE and identified by LC-MSMS. The proteins identified were compared to a list of contaminants that was established with proteins found from a mock purification (*Bacillus subtilis* 168 wild-type strain) as well as proteins recovered in more than 85% of different SPA-tagged protein purifications [1]. Proteins presented in this table have been found in two independent purifications of YlbM-SPA. Co-purified contaminating proteins are not represented. For each protein detected the average PAI (protein Abundance Index [2] is presented as an average value from the two independent experiments.

1. Delumeau O, Lecointe F, Muntel J, Guillot A, Guedon E, et al. (2011) The dynamic protein partnership of RNA polymerase in Bacillus subtilis. Proteomics 11: 2992-3001.
2. Ishihama Y, Oda Y, Tabata T, Sato T, Nagasu T, et al. (2005) Exponentially modified protein abundance index (emPAI) for estimation of absolute protein amount in proteomics by the number of sequenced peptides per protein. Mol Cell Proteomics 4: 1265-1272.
