# Supplementary material for: Constitutive Stringent Response Restores Viability of Bacillus subtilis Lacking Structural Maintenance of Chromosome Protein
Source: PLoS One. 2015 Nov 5;10(11):e0142308. doi: 10.1371/journal.pone.0142308 (PMC4634966; doi:10.1371/journal.pone.0142308)
Supplement: S4 Table — (PDF) [file pone.0142308.s007.pdf]

Strains	Colonies/uO.D.$_{600nm}$		Frequency of suppressors arising
	**LB 23°C**	**LB 37°C**	**(ratio 37°C/23°C)**
Wild type	2.5×10^8	2.5×10^8	1
Δsmc	1.3×10^8	5.1×10^4	3.9×10^{-4}
Δsmc ΔyjbM ΔywaC	9×10^7	6×10^4	6.7×10^{-4}
ΔrelA ΔyjbM ΔywaC	2×10^8	2×10^8	1
ΔsmcΔrelA ΔyjbM ΔywaC	1.9×10^7	1.5×10^3	7.8×10^{-5}

Table S4. Comparison of the number of colonies formed per OD$_{600nm}$ unit

Cells were grown to OD$_{600nm}$ between 0.3 and 0.6 in rich medium at 23°C and then spread on LB plate and incubated at 23°C or 37°C, as indicated. Number of colonies formed at 23°C or 37°C were determined after 4 or 2 days of incubation respectively. Results are the mean of two or three independent experiments.
